# Predominant Pneumococcal Serotypes in Isolates Causing Invasive Disease in a Spanish Region: An Examination of Their Association with Clinical Factors, Antimicrobial Resistance, and Vaccination Coverage

**DOI:** 10.3390/jcm14051612

**Published:** 2025-02-27

**Authors:** Laura Diab-Casares, Nuria Tormo-Palop, Fco Javier Hernández-Felices, Violeta Artal-Muñoz, Pedro Floría-Baquero, José Luis Martin-Rodríguez, Rafael Medina-González, Sonia Cortés-Badenes, Begoña Fuster-Escrivá, Ana Gil-Bruixola, José Luis López-Hontangas, Fco Javier Roig-Sena, Herme Vanaclocha-Luna, Remedio Guna-Serrano, Concepción Gimeno-Cardona

**Affiliations:** 1 Departamento de Microbiología, Consorcio Hospital General Universitario de Valencia, 46014 Valencia, Spain; lau.dicasares@gmail.com (L.D.-C.); nuriatormo274@gmail.com (N.T.-P.); fjhernandezfelices@gmail.com (F.J.H.-F.); violetaartal@gmail.com (V.A.-M.); pedrof96@hotmail.com (P.F.-B.); jl-mr@outlook.es (J.L.M.-R.); cortes_sonbad@gva.es (S.C.-B.); begonafuster@gmail.com (B.F.-E.); reme.guna@gmail.com (R.G.-S.); 2 Departamento de Microbiología, Hospital Universitario La Fe, 46026 Valencia, Spain; agilbru@hotmail.com (A.G.-B.); lopez_jlu@gva.es (J.L.L.-H.); 3 Servicio de Vigilancia y Control Epidemiológico, Dirección General de Salud Pública, Conselleria de Sanitat, Comunitat Valenciana, 46010 Valencia, Spain; see@geyseco.es (F.J.R.-S.); vanaclocha_her@gva.es (H.V.-L.); 4 Facultad de Medicina, Departamento de Microbiología, Universidad de Valencia, 46010 Valencia, Spain

**Keywords:** Comunidad Valenciana, invasive pneumococcal disease, serotypes, *Streptococcus pneumoniae*

## Abstract

**Background**: *Streptococcus pneumoniae* (*S. pneumoniae*) remains a leading cause of morbidity and mortality, particularly among vulnerable populations. This study investigates the epidemiology of pneumococcal serotypes associated with invasive pneumococcal disease (IPD) in the Comunidad Valenciana (CV) region, Spain, analysing 1587 isolates collected from 2014 to 2023. **Methods**: Serotyping and antimicrobial susceptibility testing were performed, and whole-genome sequencing was conducted on 104 isolates to explore their clonal relationships. **Results**: The most prevalent serotype was serotype 8 (17.5%), followed by serotype 3 (14.7%), with a notable increase in serotype 8 cases from 2019 onwards and a decline in serotype 19A being observed. Age distribution played a role, as serotype 8 was more frequent in individuals over ten years old. The overall recovery rate was 72%, while serotypes 3 and 15A exhibited the highest mortality rates. The vaccination coverage was highest among children under five, underscoring the need for continued surveillance to evaluate vaccine effectiveness. The antimicrobial resistance was most pronounced for erythromycin (20%) and clindamycin (16%), with serotypes 19A and 6C displaying the highest resistance levels. Whole-genome sequencing identified sequence type (ST) 53 and ST180 as the predominant STs for serotypes 8 and 3, respectively, mirroring global trends. **Conclusions**: These findings emphasize the necessity of continuous monitoring to inform vaccination policies and antimicrobial strategies, to ensure effective disease control and improved patient outcomes.

## 1. Introduction

*Streptococcus pneumoniae* (*S. pneumoniae*) is a significant pathogen due to its high morbidity and mortality, particularly in individuals at the extremes of age or those with underlying conditions that compromise their immunity. This can lead to invasive pneumococcal disease (IPD) [1,2], which is defined as the isolation of *S. pneumoniae* from sterile body fluids such as blood, cerebrospinal fluid, synovial fluid, pleural exudate, or pericardial fluid. The likelihood of a colonised individual developing IPD is closely linked to the serotype involved.

Polysaccharide and conjugate vaccines have been developed based on the most prevalent capsular serotypes associated with IPD, and demonstrate high efficacy in preventing infection and reducing transmission. The introduction of these vaccines has altered the epidemiology of *S. pneumoniae*, making continuous surveillance essential [3]. The distribution of the pneumococcal serotypes that cause invasive disease varies across different geographical regions and fluctuates over time [4], which is particularly relevant given that current vaccines target a select number of serotypes.

Another crucial aspect of pneumococcal disease is the relationship between the pathogen’s serotype and its antimicrobial resistance. Some serotypes are more frequently associated with resistance to penicillin and other antibiotics, emphasizing the need for the continuous monitoring of resistance patterns. For many years, penicillin was the first-line treatment for *S. pneumoniae* due to its strong bactericidal activity and low minimum inhibitory concentrations (MICs). However, the global prevalence of penicillin-resistant strains has increased significantly, with Spain reporting one of the highest resistance rates in Europe. For example, a study conducted in Madrid between 2007 and 2022 found that 32.3% of *S. pneumoniae* isolates were resistant to penicillin [5]. Additionally, approximately 6% of the isolates in Europe exhibit resistance to cephalosporins [6].

Macrolide resistance has also risen sharply in Spain over the past two decades, now reaching 33.2–46.8% in non-invasive strains and 20–30% in invasive strains, largely due to high antibiotic consumption [7,8]. In contrast, quinolones, which are widely used for treating non-severe pneumonia in adults, maintain relatively low resistance rates—around 3% in this geographical area [9]—likely because their use is not recommended in children, who are the primary reservoir for *S. pneumoniae*. Despite these trends, there is a notable lack of recent studies examining pneumococcal antimicrobial susceptibility in this region.

The aim of this study is to determine the epidemiology and distribution of *S. pneumoniae* serotypes that cause IPD in the Comunidad Valenciana (CV) region and to assess their prevalence. This study also explores the relationship between the serotype and clinical factors, categorising patients into those with 0–1 comorbidity and those with more than one. Additionally, it examines serotype-specific patterns of antimicrobial resistance. Furthermore, the whole-genome sequencing of a representative subset of pneumococcal isolates has been conducted to analyse clonal relationships.

## 2. Materials and Methods

### 2.1. Study Design and Data Collection

This is a retrospective longitudinal study analysing 1587 isolates of *S. pneumoniae* that were responsible for IPD in hospitals across the CV region between 2014 and 2023.

### 2.2. Sociodemographic and Clinical Data

All case data were obtained from the epidemiological surveillance system (AVE), which integrates information from three primary sources: outpatient information systems, hospital preventive medicine departments, and the microbiological surveillance network of the CV Region (RedMIVA) network [10]. RedMIVA provides real-time microbiological test results from hospitals across the region. This study collected and analysed the following variables: age, sex, serotype, infection site, comorbidities, vaccination status, antimicrobial resistance, and clinical outcomes.

### 2.3. Serotyping and Antimicrobial Sensitivity Testing

While each RedMIVA-affiliated laboratory determines its own bacteriological detection methods, they all adhere to standardised interpretation criteria.

The serotyping of pneumococcal isolates was carried out using the Neufeld capsule test at the Microbiology Service of Hospital La Fe in Valencia or at the National Microbiology Centre from 2014 to 2021. From 2022 onwards, serotyping was performed using polymerase chain reaction (PCR) followed by reverse hybridisation (S. PneumoStrip, Operon, Immuno & Molecular Diagnostics, Zaragoza, Spain) at the current reference centre for pneumococcal serotyping in the CV region, the Consorcio Hospital General Universitario de Valencia.

Antimicrobial susceptibility testing was conducted for the following antibiotics: penicillin, ceftriaxone, vancomycin, linezolid, levofloxacin, erythromycin, and clindamycin. Susceptibility was assessed in accordance with the guidelines established by the Clinical and Laboratory Standards Institute (CLSI) and the European Committee on Antimicrobial Susceptibility Testing (EUCAST).

### 2.4. Whole-Genome Sequencing

Whole-genome sequencing was performed on a representative subset of 104 pneumococcal isolates. DNA extraction was carried out using the MagNA^®^ pure Compact (Roche Diagnostics, Rotkreuz, Switzerland). DNA libraries were prepared using the Nextera XT kit and sequenced on a MiSeq platform (Illumina, San Diego, CA, USA).

### 2.5. Bioinformatics Analysis

For quality assessment and de novo assembly of sequencing data, INNUca was used, with genome assembly being performed using SPAdes. The resulting sequences were uploaded to Pathogenwatch “https://pathogen.watch/ (accessed on 27 April 2023)” in FASTA format, where multilocus sequence typing (MLST) was conducted.

### 2.6. Statistical Analysis

Categorical variables were expressed as frequencies and percentages. Differences between groups were assessed using Pearson’s chi-squared test (Chi^2^), while Fisher’s exact test was applied for 2 × 2 tables. Bonferroni correction was used for multiple comparisons, and effect sizes were calculated to aid result interpretation.

Effect sizes for categorical variables were measured using Cramér’s V and classified as follows:

- Negligible: 0.00–0.09;
- Low: 0.10–0.29;
- Medium: 0.30–0.49;
- High: ≥0.50.

A significance level of 5% (α = 0.05)^2^ was applied to all analyses.

## 3. Results

### 3.1. Demographic Characteristics

Of the 1587 cases of IPD that were analysed, 39.7% occurred in females and 60.3% occurred in males. The mean age of the patients was 61 years (±23.41), with a range spanning from 1 to 100 years. When stratified into age groups, the distribution was as follows:

- 0–4 years: 4.1%;
- 5–9 years: 1.7%;
- 10–44 years: 14.9%;
- 45–64 years: 26.4%;
- ≥65 years: 52.9%.

### 3.2. Serotype Distribution

**Figure 1:**
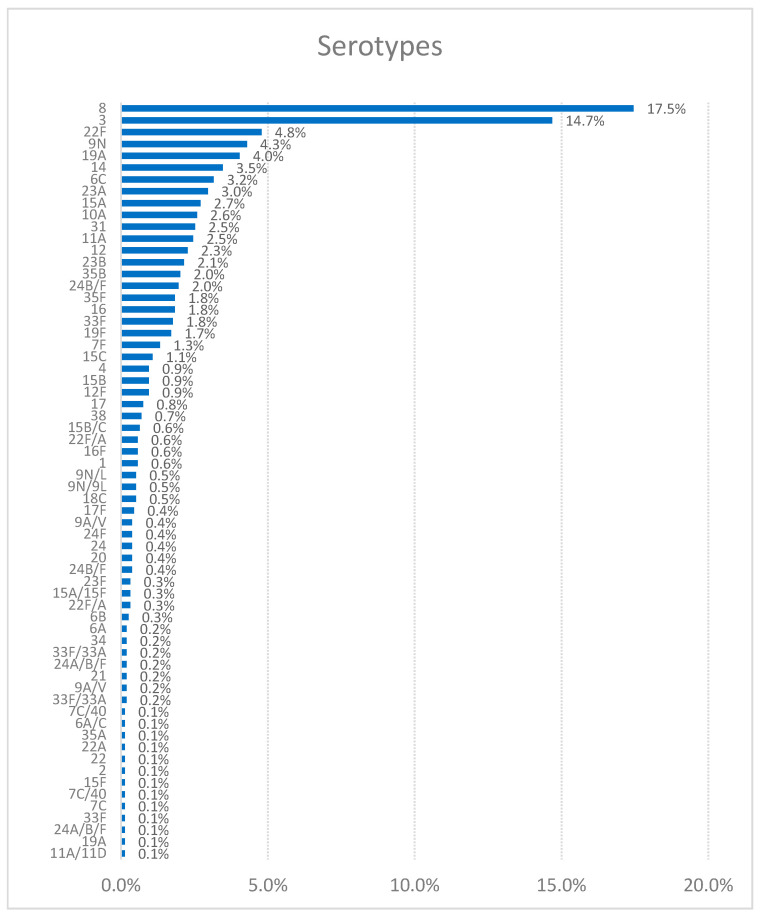
Frequency of different pneumococcal serotypes causing invasive pneumococcal disease in the Comunidad Valenciana region.

**Figure 2:**
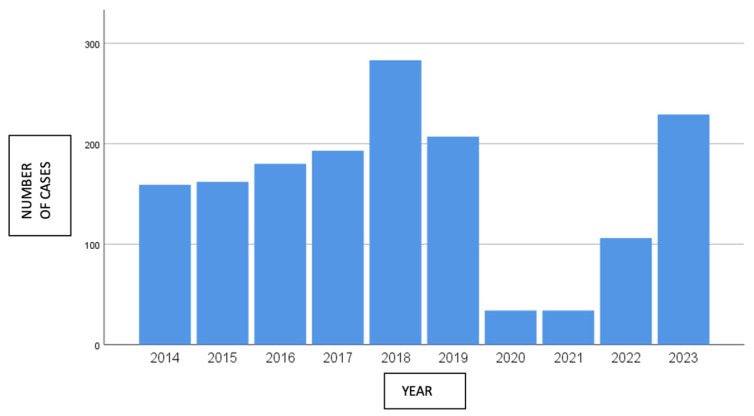
Distribution by year of pneumococcal isolates causing invasive pneumococcal disease in the Valencian Community.

A total of 100 different pneumococcal serotypes were identified. The most prevalent was serotype 8 (17.5%), followed by serotype 3 (14.7%). Other frequently detected serotypes included 22F, 9N, and 19A (each 4–5%), as well as 14, 6C, and 23A (3–4%). The remaining serotypes accounted for less than 3% of cases. Figure 1 shows the frequency of the different pneumococcal serotypes in our sample while Figure 2 analyses the distribution of IPD-producing pneumococcal isolates by year.

### 3.3. Temporal Evolution of Serotypes

Annual trends showed a decline in IPD cases in 2020, 2021, and 2022, which is likely linked to public health measures implemented during the COVID-19 pandemic (Table 1).

**Figure 3:**
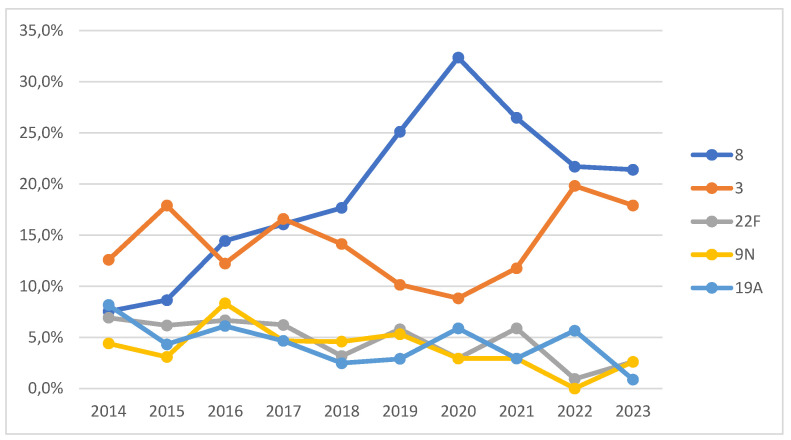
Yearly evolution of the most frequent serotypes found in the study. Each serotype is represented as a percentage (y-axis) of the total annual isolates (x-axis).

An analysis of the five most prevalent serotypes (8, 3, 22F, 9N, and 19A) revealed significant changes over time (χ^2^ = 1270.905, *p* < 0.001) (Figure 3):

- The prevalence of Serotype 8 increased markedly from 2019 onwards, peaking in 2020 at 32%;
- Serotype 19A exhibited a steady decline, exhibiting a prevalence of 8% in 2014 and falling to just 1% in 2023.

### 3.4. Age and Serotype Association

While no significant differences were observed in the serotype distribution between sexes (χ^2^ = 97.103, *p* = 0.594), notable differences were identified across age groups (χ^2^ = 518.460, *p* < 0.001 (Table 2). Specifically:

- Serotype 8 was more common in individuals over 10 years of age, particularly those within the 10–64 age range;
- Serotype 10A was most frequently detected in children aged 5–9 years.

### 3.5. Clinical Outcomes

Among the patients with known outcomes:

- 72.0% recovered;
- 16.6% died;
- 1.0% developed long-term sequelae;
- 9.8% had an unknown outcome.

**Figure 4:**
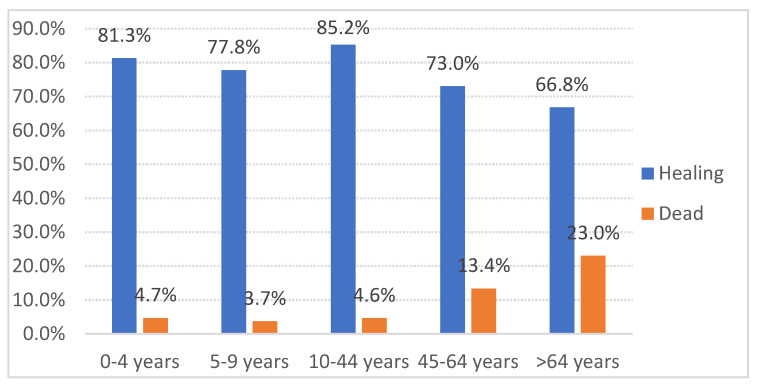
Age-related distribution of final states of the patients.

There were no differences by sex (Chi^2^ (9.410, 4) *p* 0.052), but the mortality rates varied significantly with age (χ^2^ = 70.563, *p* < 0.001), with individuals over 64 experiencing the highest fatality rate (23%) compared to younger groups (5–13%) (Figure 4).

**Figure 5:**
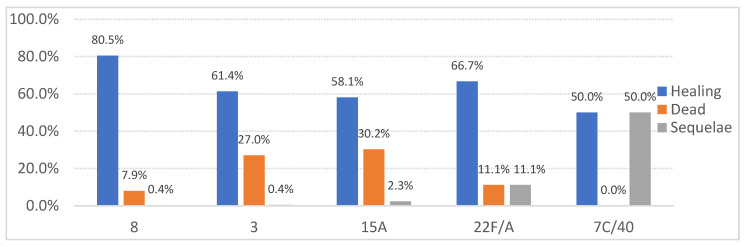
Serotype-related distribution of final invasive pneumococcal disease outcomes of the patients.

Figure 5 shows that serotype-specific analysis also indicated significant differences (Chi^2^ = 543.586, *p* < 0.001):

- Serotype 8 was associated with higher recovery rates;
- Serotypes 3 and 15A exhibited the highest mortality rates;
- Serotypes 22F/A and 7C/40 were linked to an increased incidence of sequelae, although the case numbers were low (n < 10).

**Figure 6:**
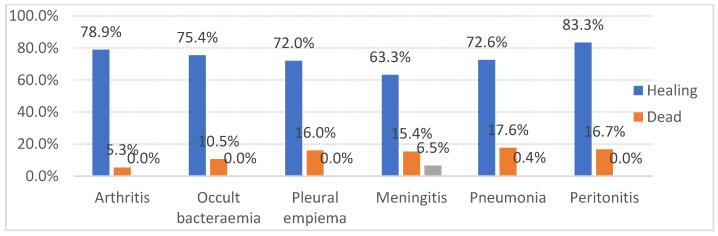
Outcome of patients by clinical form of invasive pneumococcal disease.

The mortality rates did not vary significantly across different clinical manifestations of IPD, with the pneumonia-associated cases showing the highest fatality rate (18%) and the arthritis-associated cases showing the lowest (5%). Other forms of IPD that were observed were occult bacteraemia, pleural empyema, meningitis, and peritonitis. The percentage of patients with sequelae was 7% in meningitis compared to 0.4% in pneumonia and 0% in the rest of the clinical forms (Chi^2^ (74.132, 20) *p* < 0.001) (Figure 6).

### 3.6. Prior Antibiotic Treatment

Among patients:

- 11.4% had received prior antibiotic treatment;
- 81.4% had not;
- 6.9% had an unknown treatment status.

No significant differences were found in prior antibiotic use by sex (χ^2^ = 1.095, *p* = 0.778) or age (χ^2^ = 15.713, *p* = 0.205).

### 3.7. Vaccination Coverage

- 22.7% of patients had been vaccinated;
- 72.9% were unvaccinated;
- 4.4% had an unknown vaccination status.

The vaccination rates varied significantly with age (χ^2^ = 219.207, *p* < 0.001), (Cramer’s V = 0.263) (Table 3), with the coverage being:

- 3–4 times higher in children under 5 compared to older groups;
- 8–9 times higher in children under 5 compared to those aged 45–64.

Among the vaccinated individuals, 55.3% had received the PPV23 vaccine (12.5% of the total study population).

The vaccination rate (with any pneumococcal vaccine) was 22% in patients with pneumonia who developed an IPD, with no significant differences being found compared to other clinical forms (Chi^2^ (8.601, 10) *p* 0.570). Pneumonia was the most common clinical presentation of IPD, occurring in (Table 4):

- 79.1% of unvaccinated patients;
- 75.4% of vaccinated patients (no significant difference, χ^2^ = 9.277, *p* = 0.813).

The most common serotypes by age group for vaccinated individuals are shown/depicted in Table 5.

### 3.8. Serotype and Comorbidities

Patients were classified into two groups:

- 0–1 comorbidity: 74.7%;
- ≥2 comorbidities: 25.3%.

The serotype distribution differed significantly between these groups (χ^2^ = 140.589, *p* = 0.006) (Table 6):

- Serotypes 3 and 6C were more common in patients with multiple comorbidities;
- Serotype 19A was more prevalent in those with 0–1 comorbidity.

The comorbidities that we analysed are: cardiovascular disease, chronic respiratory disease, traumatic brain injury, chronic otitis, diabetes, and HIV.

### 3.9. Antimicrobial/Antibiotic Resistance

**Figure 7:**
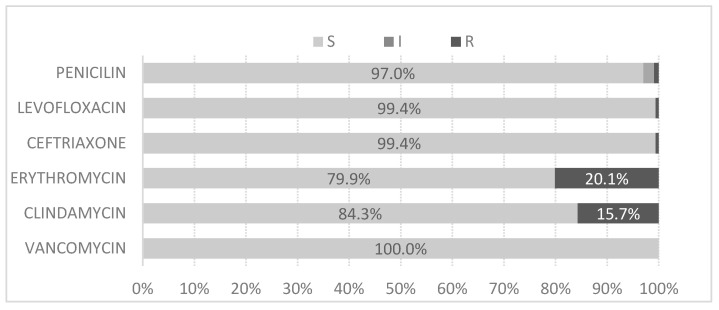
Shows the percentages of antimicrobial resistance of the invasive pneumococcal disease-producing isolates.

Among the isolates tested, the resistance rates were highest for erythromycin (20%) and clindamycin (16%). The resistance to other antibiotics was below 5% (Figure 7).

Table 7 shows serotype-specific resistance patterns:

- Serotype 8 was most common among fully susceptible isolates;
- Serotype 19A was predominant among isolates with resistance to at least one antibiotic;
- Serotype 6C showed the highest prevalence in isolates resistant to two or more antibiotics;
- Serotype 15A was frequently associated with resistance to one or two antibiotics and frequently associated with pneumococcal strains;
- Serotype 11A was most commonly found in isolates with single-drug resistance;
- Only two serotypes (19A and 6C) exhibited resistance to three antibiotics.

**Figure 8:**
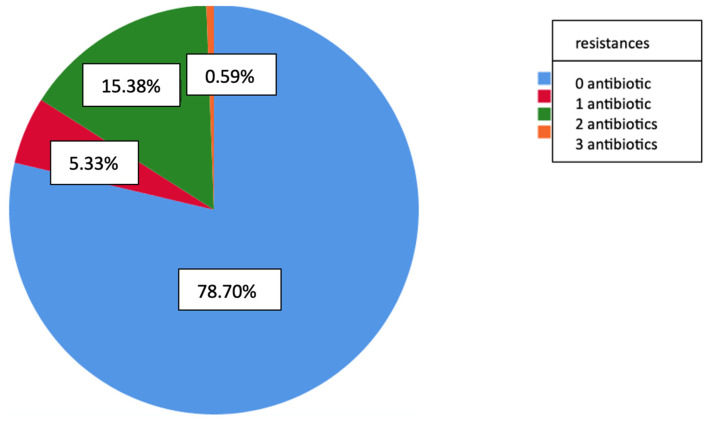
Graphical representation of resistance to none, one, or two or more antimicrobials in pneumococcal isolates producing invasive pneumococcal disease from 2014 to 2023.

A total of 78.7% of the isolates were susceptible to all six antimicrobials studied, while 5.3% exhibited resistance to a single antibiotic. Additionally, 15.4% demonstrated resistance to two antibiotics, and 0.6% were resistant to three antibiotics (Figure 8).

**Figure 9:**
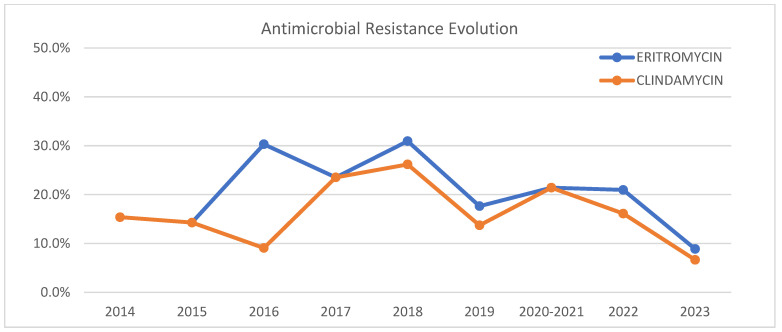
Evolution of erythromycin and clindamycin resistance of invasive pneumococcal disease-producing isolates from 2014 to 2023.

Temporal analysis showed no significant changes in resistance to erythromycin (χ^2^ = 9.886, *p* = 0.273) or clindamycin (χ^2^ = 9.390, *p* = 0.310) over the study period, as can be seen in Figure 9.

Only serotypes associated with the resistant phenotype that involved the two antibiotics with a significant resistance rate were analysed, as the remaining antibiotics had fewer than five resistant cases. The serotypes identified in the isolates that were resistant to macrolides/lincosamides were predominantly 19A, 6C, 15A, 33F, 3, and 24B/F, with prevalence rates ranging from 5% to 18%. The incidence of the first three serotypes (19A, 6C, 15A) was significantly higher in the resistant isolates compared to those susceptible to both erythromycin (χ^2^ (205.711, 46), *p* < 0.001) and clindamycin (χ^2^ (185.079, 46), *p* < 0.001). In contrast, serotypes 3 and 8 were more frequently observed in patients with isolates that were susceptible to both antibiotics (Table 8).

### 3.10. Bioinformatic Analysis

**Figure 10:**
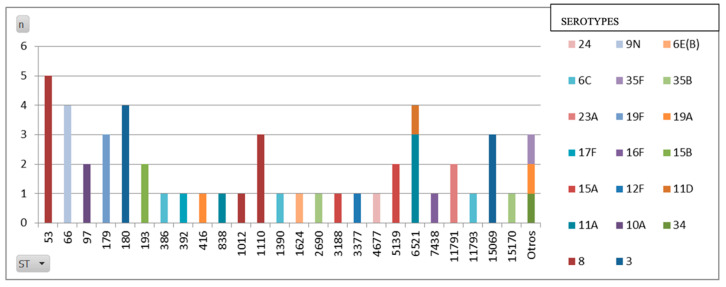
Image showing sequence types linked to serotypes.

Whole-genome sequencing identified sequence type 53 as the most prevalent ST, followed by ST180 and ST6521 (Table 9 and Figure 10).

- The serotype 8 isolates were predominantly ST53 and ST1110;
- The serotype 3 isolates were primarily ST180, with some ST15069;
- Serotype 12F was exclusively ST3377, while serotype 17F was exclusively ST392.

## 4. Discussion

In this study, the incidence of IPD by gender followed the trend observed in previous studies [11], being more frequent in males (60%) compared to females (40%). Additionally, age was found to be a significant risk factor for the development of this disease, with the mean patient age being 60 years, similar to what has been reported in other studies [12] where more than 50% of cases occurred in individuals aged 65 years or older [13]. The correlation between age and the serotype of the disease had also been documented in previous research [14].

Regarding the incidence of IPD by year, despite the availability of pneumococcal vaccines, a slightly upward trend has been observed since 2014, with a peak in 2018 of 283 cases. During 2020 and 2021, due to the reduction in the number of reported cases and prevention measures implemented during the COVID-19 pandemic, a notable decrease was recorded, with there being 34 cases per year, representing an 84% decline compared to 2019. Subsequently, an upward trend was observed from 2022, with similar figures to those before the pandemic being observed in 2023 (210 cases/year), coinciding with the relaxation of non-pharmacological measures to control the transmission of the virus.

In respect of the distribution by serotype, there are differences according to the study period and geographic region, underscoring the importance of the close monitoring of IPD. Our data are consistent with the annual report of the European Centre for Disease Prevention and Control (ECDC) [13], which indicates that serotype 8 has been the most frequent in the last six years, followed by serotypes 3, 22F, 9N, 19A, 14, 6C, and 23A. In contrast, in other regions like Serbia (2010–2018), the predominant serotypes were 3, 19F, 14, 6B, 6A, 19A, and 23F [15]. In the Canada report of 2021–2022, the most common *S. pneumoniae* serotypes in cases of invasive disease varied: in 2021, the predominant serotypes were 22F, 3, 19A, 11A, 6C, and 35B, and in 2022, the distribution changed slightly, with serotypes 22F, 3, 19A, 11A, 6C, and 35B again being prominent, but with some changes in the order of prevalence [16]. Compared to our report, it can be observed that serotype 8 is not among the most prevalent serotypes in Canada in the period 2021–2022, suggesting geographic variability in the prevalence and response to treatment of different *S. pneumoniae* serotypes.

Breaking down the five most common serotypes by year in our sample (8, 3, 22F, 9N, and 19A), our results support the phenomenon of indirect immunity following the introduction of the PCV13 vaccine in childhood vaccination in 2015 [17], especially for serotypes 3 and 19A. This explains the decrease in the incidence of serotype 19A from 2014 to 2023 (from 8% to 1%). Serotype 3 was the most common in 2014, 2015, 2017, and 2018, but from that year onwards, serotype 8 became predominant, relegating serotype 3 to second place, reflecting the effect of PCV13. Over the past 10 years, there has been an increase in the prevalence of serotypes 8 and 3, due both to the increase in IPD cases in adults and children and to the reduction in other serotypes such as 22F, 19A, 14, and 6C. Serotype 8, included in the PPSV23 vaccine, has shown a significant increase since 2019, peaking in 2020 (32%). During the COVID-19 pandemic (2020–2021), the serotype distribution was similar to that in previous years, with an increase in the incidence of serotypes 16F, 20, and 4. Other studies have noted a higher prevalence of serotypes 19A, 3, and 6C in both children and adults during this period [18].

In our study, serotypes 3 and 6C were the most frequently isolated from patients with two or more comorbidities, while serotype 8 was predominant in patients with zero or one comorbidity. Comparing these findings with those of a study conducted in CV region during the period 2007–2012, the order of the studied serotypes by their association with the highest percentage of comorbidities was as follows: 22F, 3, 1, 8, 7F, 19A, and 14 [14]. This difference could likely be attributed to the decline in the prevalence of serotypes included in the PCV-13 vaccine, reflecting the impact of its introduction. Regarding mortality by serotype, the highest case-fatality rates in our study were observed for serotypes 3 and 15A. In contrast, the study conducted in the CV region during the same period maintained serotype 3 as one of the most lethal, while serotype 19A was also found to be significant, likely as a secondary effect of the introduction of the PCV-13 vaccine.

Meanwhile, serotype 8 showed the highest cure rates, consistent with other studies [19,20], which is attributable to its greater susceptibility to standard antibiotic treatments and its lower association with severe comorbidities. These results are particularly significant, as they indicate that, in addition to being associated with severe comorbidities, these serotypes may also be linked to poorer clinical outcomes. This information is crucial for guiding clinical management and prioritizing interventions in high-risk patients.

Our data showed that 22.7% of the patients had received some form of pneumococcal vaccine, which is comparable to other studies, where vaccination rates generally range from 20–30%, depending on the geographic location and health policies [21,22].

The incidence of pneumonia leading to IPD in our study was 79.1% among unvaccinated individuals and 75.4% among vaccinated individuals. In comparison, other studies reported incidences of 65% in unvaccinated individuals and 48% in vaccinated individuals [21]. The smaller difference in IPD incidence between vaccinated and unvaccinated individuals in our study (79.1% vs. 75.4%) may be due to the high percentage of unvaccinated patients and those whose vaccination status is unknown.

A study published by the American Academy of Pediatrics found that pneumococcal conjugate vaccines (PCV-10 and PCV-13) demonstrated high effectiveness against IPD in children under 5 years of age. However, their effectiveness varies depending on the specific serotype and the vaccination schedule, suggesting that the protection provided by these vaccines is not uniform across all serotypes or age groups. The study also emphasizes that significant differences in results can arise depending on the context and population studied, which may introduce potential bias when comparing vaccinated and unvaccinated groups [23].

Additionally, an analysis of the epidemiology of invasive pneumococcal infections suggests that the incidence of IPD across different age groups may be influenced by factors such as comorbidities and vaccination coverage. In some studies, the vaccine effectiveness may be overestimated due to serotype replacement, potentially creating a misleading impression of reduced IPD rates in vaccinated populations [4].

Concerning antimicrobial susceptibility, all tested antibiotics showed less than 5% resistance, except erythromycin (20%) and clindamycin (16%). Other studies report lower resistance percentages in Morocco (2.5% for both antibiotics) [24], and higher percentages for erythromycin in Oman (28.1%) [25], Iran (71.4%) [26], and Taiwan (80%) [27]. Two of the serotypes with the highest resistance rates were 19A and 6C, possibly due to the lower metabolic cost of their polysaccharide structure, allowing them to be more capsulated and evade the immune system, persisting as nasopharyngeal carriers [28]. This behaviour could explain the higher prevalence of multidrug-resistant (MDR) lineages of serotypes 19A and 6C [18,29]. In our study, they are the only MDR serotypes that were detected. The serotypes most frequently isolated as susceptible to erythromycin and clindamycin were serotypes 3 and 8, with serotype 8 being the most commonly isolated among the isolates susceptible to all the tested antimicrobials.

Regarding the whole-genome sequencing (WGS) data, ST53 emerged as the predominant MLST in our study, mainly because it is the sequence type (ST) that is most commonly linked to serotype 8. This finding is in line with global data, as ST53 has consistently been associated with serotype 8, particularly across Europe [30]. Known as the Netherlands8-33 clone, ST53 has been one of the dominant global pneumococcal clones. Additionally, ST1110, another ST that was frequently found in our serotype 8 isolates, is less reported in the literature. However, emerging regional studies suggest that ST1110 may be locally significant.

As for serotype 3, our data revealed that ST180 was the most prevalent ST, which is consistent with global trends. ST180 is widely recognized as the primary sequence type for serotype 3 [31], and is also known as the Netherlands3-31 clone.

Differences in the prevalence of these STs could be influenced by local factors such as vaccine use, changes in public health policies, and local genetic diversity. The consistency in the results suggests that the molecular epidemiology of *S. pneumoniae*, in terms of serotypes and sequence types, follows global patterns, with specific regional variations. The findings underscore the importance of continuous surveillance to continually adapt vaccination strategies and disease control.

It is important to note that, although our findings are preliminary and have not been previously corroborated by other studies, they provide a valuable starting point for future research. Additional studies examining these associations in different populations and contexts are needed to validate our results and better understand the dynamics between specific serotypes, comorbidities, and clinical outcomes.

## 5. Conclusions

This study provides key insights into the epidemiology, serotype distribution, antimicrobial resistance, and vaccine impact of IPD in the CV region over the past decade.

Our findings confirm that serotype prevalence is dynamic, with serotype 8 emerging as the most common, while the prevalence of serotype 19A has significantly declined, likely due to the indirect effects of PCV13 vaccination. Despite its inclusion in PCV13, serotype 3 remains highly prevalent and is associated with higher mortality rates, particularly in patients with multiple comorbidities.

Age was identified as a major determinant of serotype distribution, disease severity, and vaccination rates. The older adults experienced the highest mortality rates, particularly those infected with serotype 3 and 15A. Serotype 8 was associated with better recovery rates, possibly due to its higher antibiotic susceptibility.

Antimicrobial resistance patterns remain a concern, particularly for erythromycin (20%) and clindamycin (16%), with serotypes 19A and 6C displaying the highest levels of multidrug resistance (MDR). In contrast, serotype 8 was the most frequently isolated among the fully susceptible strains.

Whole-genome sequencing revealed that ST53 (serotype 8) and ST180 (serotype 3) were the dominant STs, aligning with global trends. These findings underscore the importance of genomic surveillance to monitor emerging clones and vaccine escape variants.

Moving forward, further studies are needed to:

- Assess vaccination coverage and effectiveness in both paediatric and adult populations;
- Monitor the long-term impact of serotype replacement on disease prevalence;
- Evaluate antibiotic stewardship strategies to combat rising resistance trends.

Finally, future research should ensure that data collection is free from pandemic-related disruptions to allow for a clearer assessment of pneumococcal disease trends in a post-COVID-19 era.

## Figures and Tables

**Table 1:** Distribution of serotypes causing invasive pneumococcal disease per year in the Comunidad Valenciana region.

		YEAR											
		Total		2014		2015		2016		2017		2018	
		Count	Column N %	Count	Column N %	Count	Column N %	Count	Column N %	Count	Column N %	Count	Column N %
**SEROTYPE**	**Total**	1587	100.0%	159	100.0%	162	100.0%	180	100.0%	193	100.0%	283	100.0%
	**8**	277	17.5%	12	7.5%	14	8.6%	26	14.4%	31	16.1%	50	17.7%
	**3**	233	14.7%	20	12.6%	29	17.9%	22	12.2%	32	16.6%	40	14.1%
	**22F**	76	4.8%	11	6.9%	10	6.2%	12	6.7%	12	6.2%	9	3.2%
	**9N**	68	4.3%	7	4.4%	5	3.1%	15	8.3%	9	4.7%	13	4.6%
	**19A**	64	4.0%	13	8.2%	7	4.3%	11	6.1%	9	4.7%	7	2.5%
	**14**	55	3.5%	10	6.3%	10	6.2%	5	2.8%	7	3.6%	8	2.8%
	**6C**	50	3.2%	5	3.1%	8	4.9%	7	3.9%	3	1.6%	11	3.9%
	**23A**	47	3.0%	5	3.1%	3	1.9%	6	3.3%	4	2.1%	8	2.8%
**2019**			**2020**			**2021**		**2022**			**2023**		
**Count**	**Column N %**		**Count**	**Column N %**		**Count**	**Column N %**	**Count**	**Column N %**		**Count**	**Column N %**	
207	100.0%		34	100.0%		34	100.0%	106	100.0%		229	100.0%	
52	25.1%		11	32.4%		9	2.5%	23	21.7%		49	21.4%	
21	10.1%		3	8.8%		4	11.8%	21	19.8%		41	17.9%	
12	5.8%		1	2.9%		2	5.9%	1	0.9%		6	2.6%	
11	5.3%		1	2.9%		1	2.9%	0	0.0%		6	2.6%	
6	2.9%		2	5.9%		1	2.9%	6	5.7%		2	0.9%	
6	2.9%		1	2.9%		0	0.0%	0	0.0%		8	3.5%	
9	4.3%		0	0.0%		0	0.0%	1	0.9%		6	2.6%	
4	1.9%		0	0.0%		1	2.9%	4	3.8%		12	5.2%	

**Table 2:** Age-related distribution of serotypes causing invasive pneumococcal disease in the Comunidad Valenciana region.

		AGE											
		Total		0–4 Years		5–9 Years		10–44 Years		45–64 Years		>64 Years	
		Count	%	Count	%	Count	%	Count	%	Count	%	Count	%
**SEROTYPE**	**Total**	1587	100.0%	64	100.0%	27	100.0%	237	100.0%	419	100.0%	840	100.0%
	**8**	277	17.5%	3	4.7%	1	3.7%	53	22.4%	96	22.9%	124	14.8%
	**3**	233	14.7%	5	7.8%	6	22.2%	24	10.1%	55	13.1%	143	17.0%
	**22F**	76	4.8%	3	4.7%	2	7.4%	10	4.2%	20	4.8%	41	4.9%
	**9N**	68	4.3%	0	0.0%	0	0.0%	9	3.8%	23	5.5%	36	4.3%
	**19A**	64	4.0%	4	6.3%	1	3.7%	10	4.2%	11	2.6%	38	4.5%
	**14**	55	3.5%	3	4.7%	2	7.4%	5	2.1%	10	2.4%	35	4.2%
	**6C**	50	3.2%	1	1.6%	0	0.0%	2	0.8%	14	3.3%	33	3.9%
	**23A**	47	3.0%	3	4.7%	0	0.0%	6	2.5%	7	1.7%	31	3.7%
	**15A**	43	2.7%	2	3.1%	1	3.7%	8	3.4%	10	2.4%	22	2.6%
	**10A**	41	2.6%	2	3.1%	4	14.8%	10	4.2%	12	2.9%	13	1.5%
	**31**	40	2.5%	0	0.0%	0	0.0%	3	1.3%	11	2.6%	26	3.1%
	**11A**	39	2.5%	1	1.6%	1	3.7%	4	1.7%	9	2.1%	24	2.9%
	**12**	36	2.3%	0	0.0%	0	0.0%	7	3.0%	13	3.1%	16	1.9%
	**23B**	34	2.1%	4	6.3%	1	3.7%	5	2.1%	10	2.4%	14	1.7%
	**35B**	32	2.0%	2	3.1%	1	3.7%	3	1.3%	8	1.9%	18	2.1%
	**24B/F**	31	2.0%	4	6.3%	0	0.0%	8	3.4%	3	0.7%	16	1.9%

**Table 3:** Percentage of known vaccination according to age range in the Comunidad Valenciana region.

		Age											
		Total		≤4 Years		5–9 Years		10–44 Years		45–64 Years		>64 Years	
		N	%	N	%	N	%	N	%	N	%	N	%
**Pneumococcal vaccination**	**Total**	1587	100.0%	64	100.0%	27	100.0%	237	100.0%	419	100.0%	840	100.0%
	**Unknown**	70	4.4%	2	3.1%	0	0.0%	7	3.0%	28	6.7%	33	3.9%
	**No**	1157	72.9%	11	17.2%	6	22.2%	162	68.4%	353	84.2%	625	74.4%
	**Yes**	360	22.7%	51	79.7%	21	77.8%	68	28.7%	38	9.1%	182	21.7%

**Table 4:** Percentage of known vaccination according to the clinical form.

		Antipneumococcal Vaccine							
		Total		Unknown		No		Yes	
		Count	Column N %	Count	Column N %	Count	Column N %	Count	Column N %
**Clinical form infection**	**Total**	1585	100.0%	70	100.0%	1157	100.0%	358	100.0%
	Pneumonia	1242	78.4%	57	8.4%	915	79.1%	270	75.4%
	Meningitis	169	10.7%	6	8.6%	122	10.5%	41	11.5%
	Occult bacteraemia	114	7.2%	4	5.7%	75	6.5%	35	9.8%
	Pleural empyema	25	1.6%	2	2.9%	18	1.6%	5	1.4%
	Arthritis	19	1.2%	1	1.4%	13	1.1%	5	1.4%
	Peritonitis	12	0.8%	0	0.0%	11	1.0%	1	0.3%
	Endocarditis	3	0.2%	0	0.0%	2	0.2%	1	0.3%
	Pericarditis	1	0.1%	0	0.0%	1	0.1%	0	0.0%

**Table 5:** Serotypes found by vaccinated age group.

		0–4			5–9			10–44			45–64			>64	
		Count	Column N %		Count	Column N %		Count	Column N %		Count	Column N %		Count	Column N %
**SEROTYPE**	**Total**	51	100.0%	**Total**	21	100.0%	**Total**	68	100.0%	**Total**	38	100.0%	**Total**	182	100.0%
	**33F**	4	7.8%	**3**	5	23.8%	**24B/F**	7	10.3%	**8**	4	10.5%	**3**	24	13.2%
	**24B/F**	4	7.8%	**10A**	4	19.0%	**10A**	6	8.8%	**3**	4	10.5%	**8**	23	12.6%
	**3**	3	5.9%	**24B/F**	2	9.5%	**15A**	6	8.8%	**15A**	3	7.9%	**11A**	12	6.6%
	**19F**	3	5.9%	**22F**	1	4.8%	**3**	4	5.9%	**22F**	2	5.3%	**35F**	9	4.9%
	**19A**	3	5.9%	**14**	1	4.8%	**19A**	4	5.9%	**10A**	2	5.3%	**9N**	8	4.4%
	**8**	3	5.9%	**8**	1	4.8%	**8**	3	4.4%	**31**	2	5.3%	**6C**	8	4.4%
	**22F**	3	5.9%	**35B**	1	4.8%	**23A**	3	4.4%	**19F**	2	5.3%	**23A**	8	4.4%
	**23A**	2	3.9%	**33F**	1	4.8%	**15C**	3	4.4%	**9N/L**	2	5.3%	**19A**	7	3.8%
	**14**	2	3.9%	**24A/B/F**	1	4.8%	**33F**	3	4.4%	**6C**	1	2.6%	**14**	7	3.8%
	**35B**	2	3.9%	**23B**	1	4.8%	**16**	3	4.4%	**23B**	1	2.6%	**35B**	6	3.3%
	**22F/A**	2	3.9%	**19A**	1	4.8%	**38**	3	4.4%	**35B**	1	2.6%	**24B/F**	6	3.3%
	**15B/C**	2	3.9%	**17F**	1	4.8%	**24**	3	4.4%	**16**	1	2.6%	**22F**	5	2.7%
	**15A**	2	3.9%	**15A**	1	4.8%	**23B**	2	2.9%	**7F**	1	2.6%	**31**	5	2.7%
	**10A**	2	3.9%	**23F**	0	0.0%	**31**	2	2.9%	**35F**	1	2.6%	**15A**	4	2.2%
	**23B**	1	2.0%	**11A**	0	0.0%	**15B/C**	2	2.9%	**33F**	1	2.6%	**16**	4	2.2%
	**15B**	1	2.0%	**1**	0	0.0%	**22F**	1	1.5%	**24B/F**	1	2.6%	**23B**	4	2.2%

**Table 6:** Relationships between the number of comorbidities and the serotype.

		Comorbidities					
		Total		0–1		>1	
		Count	%	Count	%	Count	%
**SEROTYPE**	**Total**	1587	100.0%	1185	100.0%	402	100.0%
	**8**	277	17.5%	211	17.8%	66	16.4%
	**3**	233	14.7%	160	13.5%	73	18.2%
	**22F**	76	4.8%	55	4.6%	21	5.2%
	**9N**	68	4.3%	53	4.5%	15	3.7%
	**19A**	64	4.0%	57	4.8%	7	1.7%
	**14**	55	3.5%	45	3.8%	10	2.5%
	**6C**	50	3.2%	31	2.6%	19	4.7%
	**23A**	47	3.0%	32	2.7%	15	3.7%
	**15A**	43	2.7%	32	2.7%	11	2.7%
	**10A**	41	2.6%	32	2.7%	9	2.2%
	**31**	40	2.5%	30	2.5%	10	2.5%
	**11A**	39	2.5%	24	2.0%	15	3.7%
	**12**	36	2.3%	30	2.5%	6	1.5%
	**23B**	34	2.1%	28	2.4%	6	1.5%
	**35B**	32	2.0%	25	2.1%	7	1.7%
	**24B/F**	31	2.0%	22	1.9%	9	2.2%

**Table 7:** Relationships between serotypes and number of antimicrobial resistances.

		Antimicrobial Resistance									
		Total		0 Antibotics		1 Antibiotics		2 Antibiotics		3 Antibotics	
		Count	Column N %	Count	Column N %	Count	Column N %	Count	Column N %	Count	Column N %
**SEROTYPES**	**Total**	338	100.0%	266	100.0%	18	100.0%	52	100.0%	2	100.0%
	**8**	79	23.4%	77	28.9%	0	0.0%	2	3.8%	0	0.0%
	**3**	49	14.5%	44	16.5%	2	11.1%	3	5.8%	0	0.0%
	**19A**	16	4.7%	4	1.5%	3	16.7%	8	15.4%	1	50.0%
	**9N**	15	4.4%	15	5.6%	0	0.0%	0	0.0%	0	0.0%
	**22F**	14	4.1%	14	5.3%	0	0.0%	0	0.0%	0	0.0%
	**14**	13	3.8%	8	3.0%	2	11.1%	3	5.8%	0	0.0%
	**6C**	10	3.0%	1	0.4%	1	5.6%	7	13.5%	1	50.0%
	**9N/9L**	8	2.4%	6	2.3%	0	0.0%	2	3.8%	0	0.0%
	**31**	8	2.4%	8	3.0%	0	0.0%	0	0.0%	0	0.0%
	**23B**	8	2.4%	8	3.0%	0	0.0%	0	0.0%	0	0.0%
	**15A**	8	2.4%	1	0.4%	2	11.1%	5	9.6%	0	0.0%
	**16**	7	2.1%	6	2.3%	0	0.0%	1	1.9%	0	0.0%
	**11A**	7	2.1%	3	1.1%	3	16.7%	1	1.9%	0	0.0%
	**33F**	6	1.8%	0	0.0%	0	0.0%	6	11.5%	0	0.0%
	**22F/A**	6	1.8%	6	2.3%	0	0.0%	0	0.0%	0	0.0%
	**12F**	6	1.8%	4	1.5%	0	0.0%	2	3.8%	0	0.0%
	**10A**	6	1.8%	6	2.3%	0	0.0%	0	0.0%	0	0.0%
	**35F**	5	1.5%	5	1.9%	0	0.0%	0	0.0%	0	0.0%
	**35B**	5	1.5%	4	1.5%	1	5.6%	0	0.0%	0	0.0%
	**23A**	5	1.5%	5	1.9%	0	0.0%	0	0.0%	0	0.0%
	**9A/V**	4	1.2%	4	1.5%	0	0.0%	0	0.0%	0	0.0%
	**24B/F**	4	1.2%	0	0.0%	0	0.0%	4	7.7%	0	0.0%
	**15B/C**	4	1.2%	4	1.5%	0	0.0%	0	0.0%	0	0.0%
	**7F**	3	0.9%	3	1.1%	0	0.0%	0	0.0%	0	0.0%
	**7C/40**	3	0.9%	3	1.1%	0	0.0%	0	0.0%	0	0.0%
	**4**	3	0.9%	2	0.8%	1	5.6%	0	0.0%	0	0.0%
	**24F**	3	0.9%	0	0.0%	0	0.0%	3	5.8%	0	0.0%
	**19F**	3	0.9%	2	0.8%	0	0.0%	1	1.9%	0	0.0%
	**16F**	3	0.9%	3	1.1%	0	0.0%	0	0.0%	0	0.0%
	**38**	2	0.6%	2	0.8%	0	0.0%	0	0.0%	0	0.0%
	**33F/33A**	2	0.6%	0	0.0%	0	0.0%	2	3.8%	0	0.0%
	**24A/B/F**	2	0.6%	1	0.4%	0	0.0%	1	1.9%	0	0.0%
	**18C**	2	0.6%	1	0.4%	1	5.6%	0	0.0%	0	0.0%
	**17F**	2	0.6%	2	0.8%	0	0.0%	0	0.0%	0	0.0%
	**15B**	2	0.6%	2	0.8%	0	0.0%	0	0.0%	0	0.0%
	**12**	2	0.6%	2	0.8%	0	0.0%	0	0.0%	0	0.0%
	**11A/11D**	2	0.6%	2	0.8%	0	0.0%	0	0.0%	0	0.0%
	**1**	2	0.6%	2	0.8%	0	0.0%	0	0.0%	0	0.0%
	**6A/C**	1	0.3%	0	0.0%	1	5.6%	0	0.0%	0	0.0%
	**35A**	1	0.3%	1	0.4%	0	0.0%	0	0.0%	0	0.0%
	**27**	1	0.3%	1	0.4%	0	0.0%	0	0.0%	0	0.0%
	**24A**	1	0.3%	1	0.4%	0	0.0%	0	0.0%	0	0.0%
	**21**	1	0.3%	1	0.4%	0	0.0%	0	0.0%	0	0.0%
	**18B/C**	1	0.3%	1	0.4%	0	0.0%	0	0.0%	0	0.0%
	**17**	1	0.3%	1	0.4%	0	0.0%	0	0.0%	0	0.0%
	**15C**	1	0.3%	0	0.0%	1	5.6%	0	0.0%	0	0.0%
	**10A/10D**	1	0.3%	0	0.0%	0	0.0%	1	1.9%	0	0.0%

**Table 8:** Relationships between serotype-resistance and erythromycin and clindamycin.

% of RESISTANT Serotypes Out of the Total Number of Resistant Cases		Erithromycin_Resistance		Clindamycin_Resistance	
		Count	Column N %	Count	Column N %
**SEROTYPE**	**Total**	68	100.0%	53	100.0%
	**19A**	12	17.6%	8	15.1%
	**6C**	9	13.2%	8	15.1%
	**15A**	7	10.3%	5	9.4%
	**33F**	6	8.8%	6	11.3%
	**3**	5	7.4%	3	5.7%
	**24B/F**	4	5.9%	4	7.5%
	**11A**	3	4.4%	2	3.8%
	**14**	3	4.4%	2	3.8%
	**24F**	3	4.4%	3	5.7%
	**12F**	2	2.9%	2	3.8%
	**33F/33A**	2	2.9%	2	3.8%
	**8**	2	2.9%	2	3.8%
	**9N/9L**	2	2.9%	2	3.8%
	**10A/10D**	1	1.5%	1	1.9%
	**15C**	1	1.5%	0	0.0%
	**16**	1	1.5%	1	1.9%
	**18C**	1	1.5%	0	0.0%
	**19F**	1	1.5%	1	1.9%
	**24A/B/F**	1	1.5%	1	1.9%
	**4**	1	1.5%	0	0.0%
	**6A/C**	1	1.5%	0	0.0%

**Table 9:** Sequence types linked to serotypes.

Serotypes	N.º	ST	N.º	ST	N.º	ST
3	4	180	3	15,069		
6C	1	386	1	1390	1	11,793
6E(B)	1	1624				
8	5	53	1	1012	3	1110
10A	2	97				
11A	1	838	3	6521		
11D	1	6521				
12F	1	3377				
15A	1	3188	2	5139		
16F	1	7438				
17F	1	392				
19A	1	416	1	NC		
19F	3	179				
23A	2	11,791				
35B	1	2690	1	15,170		
35F						

## Data Availability

Data are contained within the article.
